# Continuous Monitoring of pH and Blood Gases Using Ion-Sensitive and Gas-Sensitive Field Effect Transistors Operating in the Amperometric Mode in Presence of Drift

**DOI:** 10.3390/bios9010044

**Published:** 2019-03-18

**Authors:** Shahriar Jamasb

**Affiliations:** Department of Biomedical Engineering, Hamedan University of Technology, Hamedan 65169-13733, Iran; jamasb@hut.ac.ir; Tel.: +98-81-3841-1511

**Keywords:** amperometric, continuous monitoring, current mode, drift, GasFET, ISFET, Instability, pH

## Abstract

Accurate and cost-effective integrated sensor systems for continuous monitoring of pH and blood gases continue to be in high demand. The capacity of ion-selective and Gas-sensitive field effect transistors (FETs) to serve as low-power sensors for accurate continuous monitoring of pH and blood gases is evaluated in the amperometric or current mode of operation. A stand-alone current-mode topology is employed in which a constant bias is applied to the gate with the drain current serving as the measuring signal. Compared with voltage-mode operation (e.g., in the feedback mode in ion-selective FETs), current-mode topologies offer the advantages of small size and low power consumption. However, the ion-selective FET (ISFET) and the Gas-sensitive FET (GasFET) exhibit a similar drift behavior, imposing a serious limitation on the accuracy of these sensors for continuous monitoring applications irrespective of the mode of operation. Given the slow temporal variation associated with the drift characteristics in both devices, a common post-processing technique that involves monitoring the variation of the drain current over short intervals of time can potentially allow extraction of the measuring signal in presence of drift in both sensor types. Furthermore, in the amperometric mode the static sensitivity of a FET-based sensor, given by the product of the FET transconductance and the sensitivity of the device threshold voltage to the measurand concentration, can be increased by adjusting the device design parameters. Increasing the sensitivity, while of interest in its own right, also enhances the accuracy of the proposed method. Rigorous analytical validation of the method is presented for GasFET operation in the amperometric mode. Moreover, the correction algorithm is verified experimentally using a Si_3_N_4_-gate ISFET operating in the amperometric mode to monitor pH variations ranging from 3.5 to 10.

## 1. Introduction

The health care system will encounter a major challenge in the near future due to the ageing of the population. Biomedical sensors will potentially be able to address this challenge by providing the ability to monitor important body functions as part of preventive medical practices or by serving as an enabling technology for telemedicine to reduce healthcare costs. The pH-sensitive ion-selective field effect transistor (ISFET) and the FET-based oxygen-sensitive gas sensor e.g., with biocompatible hydrogel Nafion, represent important FET-based biomedical sensors. Monitoring of plasma carbon dioxide level can be accomplished by utilizing the change in pH coinciding with the change in the liquid-phase CO_2_ concentration as a result of formation or dissociation of carbonic acid. In particular, the changes in pH corresponding to changes in the partial pressure of CO_2_ can be measured using an ISFET.

The ISFET was introduced in 1970 by Bergveld [1] as a solid-state sensor merging the chemical sensitivity of a membrane with the amplifying capability of a field effect transistor. The origin of FET-based gas sensors dates back to the introduction of the hydrogen-sensitive gas sensor in 1975 using palladium as a catalytic metal forming the conductive gate of a metal-oxide-semiconductor FET or MOSFET [2]. FET-based sensors benefit from the advantages of integrated circuit (IC) technology, namely miniature size, ruggedness, and low manufacturing cost as compared with sensors relying on the chemical electrode technology e.g., the glass pH-meter. Although sufficiently small conventional electrodes are available for in vivo applications, they tend to be relatively fragile and expensive, thereby precluding their widespread use for biomedical applications. FET-based sensors, on the other hand, are compatible with the complementary metal oxide semiconductor (CMOS) IC technology, which readily permits batch fabrication of FET-based biosensors sufficiently small for incorporation into catheter tips.

Biosensors generally employ potentiometric or amperometric modes of electrochemical transduction to convert a given biological interaction into an electrical signal. The response of a potentiometric biosensor is obtained by measuring the potential difference arising from changes in the analyte concentration between the output electrode of the transducer and a reference electrode. In contrast, the response of an amperometric biosensor corresponds to current versus concentration variations. The response of the FET-based biosensors can be represented either by variations of the threshold voltage or those of the drain current resulting from the changes in the analyte concentration. For example, in the popular potentiometric mode of ISFET operation, commonly known as the feedback mode, a constant drain current is established through application of negative feedback via the reference electrode, allowing measurement of the equilibrium interfacial potential which depends on the ion concentration. In the current mode of sensor operation, however, the FET-based sensor can be operated as a stand-alone device by applying a constant potential to the gate electrode, sparing the need for integration of several operational amplifiers, with their high transistor counts, to obtain a fixed drain current. This permits a significant reduction in the size and complexity of the sensor system. In addition, with the omission of power-hungry operational amplifiers, the current mode of operation represents a viable option for low-power applications.

However, regardless of whether a current-mode or a voltage-mode topology is employed, threshold voltage instability, also known as drift, has imposed a significant limitation on utilization of the ISFET and the GasFET for continuous monitoring of pH and blood gases. In both devices, drift is commonly manifested as a slow temporal variation in the threshold voltage and, consequently, in the drain current of FET while the quantity of the measurand i.e., concentration of the ion or partial pressure of the gas remains fixed. This phenomenon precludes utilization of ISFET and the GasFET for in vivo continuous monitoring due to the narrow physiological range associated with the plasma pH and blood gases. Continuous monitoring of blood pH during surgery demands stabilities better than 0.002 pH/h, which is equivalent to a maximum drift rate of 0.12 mV/h assuming that the ISFET exhibits an ideal Nernstian response with a slope of −61.8 mV/pH at a temperature of 37 °C, the normal body temperature. The rate of long-term drift in pH-sensitive ISFETs at pH = 7 is typically on the order of several tenths of a millivolt per hour. To date a quantitative physical model for drift in GasFETs has not been proposed. However, a quantitative physical model accounting for the dynamics of the nonlinear drift behavior exhibited by Si_3_N_4_-gate and Al_2_O_3_-gate pH-sensitive ISFETs [3,4] has been proposed.

Adopting a suitable strategy to counteract sensor drift in the amperometric mode can potentially allow realization of low-power sensor operation with improved accuracy. Unlike approaches requiring device design modifications [5,6] or circuit-based approaches [7], analytical methods for correction of ISFET drift involving post-processing of the measuring signal [8,9] cannot compensate drift in real time. Nevertheless, simple analytical approaches represent cost-effective solutions to the sensor drift problem, which can be readily implemented within the framework of healthcare computing systems. The performance of gas sensors is also limited by sensor drift imposing high recalibration costs [10]. Analytical solutions proposed to address gas sensor drift include relatively simple pre-processing techniques involving baseline manipulation, which are often limited by the non-deterministic nature of drift [11]. More sophisticated and computationally intensive pre-processing methods relying on sensor signal processing in the frequency domain, such as feature extraction based on discrete wavelet transform, can also effectively filter out low-frequency drift [12]. While attuning methods such as orthogonal signal correction [10] attempt to estimate drift components directly from the training data, they require a large set of training data characterizing the drift behavior. In this work, a simple post-processing method for correction of drift in ISFETs and GasFETs operating in the amperometric mode is presented, which relies on windowing of the drain current over short sampling time intervals. Analytical validation of the method is presented for GasFET operation in the current mode to demonstrate the potential utility of this technique for correction of GasFET drift, given the similarity between the drift behavior of the ISFET and the GasFET. In addition, the proposed method is verified experimentally by monitoring step changes in pH in the 3.5–10 range using a Si_3_N_4_-gate ISFET biased in the triode region.

## 2. Materials and Methods

In this section, the proposed post-processing method for correction of drift is analytically validated for a GasFET. The analytical approach followed herein is also applicable to an ISFET by considering the sensitivity of the threshold voltage to the concentration of the given ion. In addition, the proposed method for correction of drift is verified by conducting a pH monitoring experiment using a Si_3_N_4_-gate pH-sensitive ISFET operating in the amperometric mode. The experimental details concerning device fabrication, characterization of ISFET drift and sensitivity, and the experimental procedure followed for monitoring step changes in pH are also presented.

### 2.1. Analytical Method for Correction of Drift

In a GasFET biased in the amperometric mode, the differential of the drain current can be expressed as the sum of the differential of the measuring signal, $dI_{D_{\mathrm{Gas}}}$ and the differential of the drain current arising from drift, $dI_{D_{Drift}}$:

(1) $$dI_{D}=dI_{D_{\mathrm{Gas}}}+dI_{D_{Drift}}$$

where $dI_{D_{\mathrm{Gas}}}$ represents the differential change in the drain current resulting from the changes in the partial pressure of the gas e.g., O_2_. Over sufficiently short intervals of time, Δt, $dI_{D_{\mathrm{Gas}}}$ and $dI_{D_{Drift}}$ are given by

(2) $$dI_{D_{\mathrm{Gas}}}=\frac{\partial I_{D}}{\partial P_{G}}\cdot dP_{G}≅\frac{\partial I_{D}}{\partial P_{G}}\cdot \frac{dP_{G}}{dt}\cdot \Delta t$$

(3) $$dI_{D_{Drift}}≅\frac{\partial I_{D}}{\partial t}\cdot \Delta t$$

where $P_{G}$ represents the partial pressure of the gas. Given the relatively low value of the long-term drift rate in a typical GasFET, the variation in the drain current due to drift $\frac{\partial I_{D}}{\partial t}$ can become negligible for a GasFET exhibiting a high amperometric sensitivity $\frac{\partial I_{D}}{\partial P_{G}}$ or experiencing a high rate of change in the partial pressure of the gas, $\frac{dP_{G}}{dt}$. That is, given

(4) $$\frac{\partial I_{D}}{\partial t}\ll \frac{\partial I_{D}}{\partial P_{G}}\cdot \frac{dP_{G}}{dt}$$

Equation (1) can be written as

(5) $$dI_{D}≅dI_{D_{\mathrm{Gas}}}$$

In the amperometric mode, the GasFET can be biased by applying fixed voltages $V_{GS}$ and $V_{DS}$ to the gate and drain electrodes respectively, with the source electrode grounded. For a GasFET biased in the triode region of operation, the drain current is given by [13]

(6) $$I_{D_{lin}}=\left(\mu C_{ins}\frac{W}{L}\right)\left[\left(V_{GS}-V_{TH_{GasFET}}\right)V_{DS}-\frac{1}{2}V_{DS}^{2}\right]$$

where μ represents the surface mobility of electrons in an *n*-channel GasFET, $C_{ins}$ denotes the gate insulator capacitance per unit area, $V_{TH_{GasFET}}$ designates the threshold voltage of the GasFET, and finally W and L are the width and the length of the GasFET, respectively. The threshold voltage of the GasFET can be expressed as [13]

(7) $$V_{TH_{GasFET}}=V_{FB0}-\Delta \psi -\frac{Q_{D}}{C_{ins}}-\frac{Q_{I}}{C_{ins}}+2\phi_{F}$$

where $\phi_{F}$ designates the Fermi potential determined by the bulk doping concentration, Δψ represents the changes in the threshold voltage resulting from absorption of gas atoms at the metal–insulator interface, and $V_{FB0}$ denotes the flatband voltage of the MOS system, which depends on the dopant density in the semiconductor, as well as the characteristic work function of the specific metal used in a metal-based GasFET, e.g., palladium in the Pd-MOS of Lundstrom [2].

To derive the current-mode response of the GasFET to variations in the partial pressure of the gas, the amperometric sensitivity of the device, $S_{A}$, can be determined as

(8) $$S_{A}=\frac{\partial I_{D}}{\partial P_{G}}=\left(\frac{\partial I_{D}}{\partial V_{TH_{GasFET}}}\right)\left(\frac{\partial V_{TH_{GasFET}}}{\partial P_{G}}\right)$$

Noting the definition of the FET transconductance [13], $g_{m}=\frac{\partial I_{D}}{\partial V_{GS}}=-\frac{\partial I_{D}}{\partial V_{TH_{GasFET}}}$, Equation (8) can be written as

(9) $$S_{A}=\frac{\partial I_{D}}{\partial P_{G}}=-g_{m}\left(\frac{\partial V_{TH_{GasFET}}}{\partial P_{G}}\right)$$

In a metal-based work function gas sensor such as the Pd-MOS, the sensitivity of the threshold voltage to the gas concentration, $\frac{\partial V_{TH_{GasFET}}}{\partial P_{G}}$ can be modeled based on the Sieverts’ law. Specifically, for a diatomic gas, $G_{2}$, if the association and dissociation of the gas atoms represent the only surface reactions occurring, we can write:

(10) $$G_{2}\overset{\leftarrow }{\to }2G$$

where G represents the absorbed gas atom. The equilibrium constant for the reaction is given by $K_{eq}={\left[G\right]}^{2}/\left[G_{2}\right]$, where $\left[G_{2}\right]$ represents the molar concentration in moles/m^3^ of the diatomic gas and [G] denotes the concentration of the absorbed gas atoms in moles/m^2^. Therefore, assuming validity of the ideal gas law $P_{G}=N_{av}kT\left[G_{2}\right]$ with $N_{av}$, *k*, and *T* denoting the Avogadro’s number, the Boltzman’s constant, and the absolute temperature, respectively, [G] can be expressed as:

(11) $$\left[G\right]=\sqrt{\frac{K_{eq}P_{G}}{N_{av}kT}}$$

The contribution to the threshold voltage of the surface dipole potential Δψ resulting from absorption of gas atoms at the interface is given by [2]

(12) $$\Delta \psi =\frac{N_{s}p}{\epsilon_{0}}$$

where $N_{s}$ designates the area density in m^−2^ of the gas atoms absorbed on the surface of the metal at the metal–insulator interface, p represents the dipole moment of the absorbed gas atom, and $\epsilon_{0}$ is the permittivity of vacuum. $N_{s}$, in turn, can be estimated as

(13) $$N_{s}=N_{av}\left[G\right]$$

The maximum value of $N_{s}$ is on the order of 10^19^ m^−2^, which corresponds to a metal–insulator interface with near saturation of surface absorption sites, e.g., an interface with one hydrogen atom per palladium atom in Pd-MOS. Substituting the expression given by Equation (11) for [G] into (13) yields

(14) $$N_{s}=\sqrt{\frac{N_{av}K_{eq}P_{G}}{kT}}$$

Substituting Equation (14) into Equation (12), the expression for Δψ will be given by

(15) $$\Delta \psi =K_{G}\sqrt{P_{G}}$$

where $K_{G}=\left(\frac{p}{\epsilon_{0}}\right)\sqrt{\frac{N_{av}K_{eq}}{kT}}$ is a temperature-dependent proportionality constant. Accordingly, using Equation (15), Equation (9) can be rewritten as:

(16) $$S_{A}=-g_{m}\left(\frac{\partial V_{TH_{GasFET}}}{\partial P_{G}}\right)=-g_{m}\left(\frac{\partial V_{TH_{GasFET}}}{\partial \Delta \psi }\right)\left(\frac{\partial \Delta \psi }{\partial P_{G}}\right)=-g_{m}\left(-1\right)\left(\frac{K_{G}}{2\sqrt{P_{G}}}\right)=\frac{g_{m}K_{G}}{2\sqrt{P_{G}}}$$

Therefore, the transfer characteristics of the GasFET are nonlinear with the amperometric sensitivity depending on the partial pressure of the gas and the operating point of the FET. For a GasFET operating in the linear region of the I-V characteristics, the sensitivity $S_{A_{lin}}$ can be written in terms of the device transconductance $g_{m_{lin}}=\frac{\partial I_{D_{lin}}}{\partial V_{GS}}=\mu C_{ins}\frac{W}{L}V_{DS}$ as:

(17) $$S_{A_{lin}}=\frac{g_{m_{lin}}K_{G}}{2\sqrt{P_{G}}}=\left(\frac{K_{G}}{2}\right)\left(\mu C_{ins}\frac{W}{L}\right)\left(\frac{V_{DS}}{\sqrt{P_{G}}}\right)$$

If we let Δt→dt, by substituting Equation (2) into (5) and replacing $\frac{\partial I_{D}}{\partial P_{G}}$ by $S_{A_{lin}}$, we will have:

(18) $$dI_{D}≅S_{A_{lin}}dP_{G}$$

Integrating Equation (18) over the interval Δt we obtain

(19) $$\int_{t}^{t+\Delta t}dI_{D}≅\overset{P_{G}+\Delta P_{G}}{\underset{P_{G}}{\int }}S_{A_{lin}}dP_{G}{}^{\prime }$$

Substituting the expression for $S_{A_{lin}}$ given by Equation (17) into Equation (19) yields

(20) $$\Delta I_{D_{lin}}=I_{D}\left(t+\Delta t\right)-I_{D}\left(t\right)=K_{G}g_{m_{lin}}{\left[\sqrt{P_{G}{}^{\prime }}\right]}_{P_{G}}^{P_{G}+\Delta P_{G}}=K_{G}g_{m_{lin}}\left(\sqrt{P_{G}+\Delta P_{G}}-\sqrt{P_{G}}\right)$$

Over short intervals of time, $\Delta P_{G}\ll P_{G}$, ${\left[\sqrt{P_{G}{}^{\prime }}\right]}_{P_{G}}^{P_{G}+\Delta P_{G}}$ can be written as:

(21) $$\sqrt{P_{G}+\Delta P_{G}}-\sqrt{P_{G}}=\sqrt{P_{G}}\left[{\left(1+\frac{\Delta P_{G}}{P_{G}}\right)}^{1/2}-1\right]≅\sqrt{P_{G}}\left[\left(1+\frac{\Delta P_{G}}{2P_{G}}\right)-1\right]=\frac{\Delta P_{G}}{2\sqrt{P_{G}}}$$

Using Equation (21), Equation (20) yields $\Delta P_{G}$ as

(22) $$\Delta P_{G}=\frac{2\Delta I_{D_{lin}}\sqrt{P_{G}}}{K_{G}g_{m_{lin}}}$$

Consequently, over sufficiently small sampling intervals, Δt, the variation of the drain current in the triode region basically corresponds to changes in the $P_{G}$, which allows extraction of the GasFET response in presence of drift over a given time interval ($t_{0}$,$t_{n}$) through summation of $\Delta I_{D_{lin}}$ values. Specifically, based on Equation (22) the change in $P_{G}$ can be expressed as

(23) $$P_{G}\left(t_{n}\right)-P_{G}\left(t_{0}\right)=\left(\frac{2}{K_{G}g_{m_{lin}}}\right)\overset{k=n}{\underset{k=0}{\sum }}\left[\Delta I_{D_{lin}{}_{\left(t_{k}\to t_{k+1}\right)}}\sqrt{P_{G}\left(t_{k}\right)}\right]$$

where $\Delta I_{D_{lin}{}_{\left(t_{k}\to t_{k+1}\right)}}=I_{D_{lin}}\left(t_{k+1}\right)-I_{D_{lin}}\left(t_{k}\right)$ denotes the change in the measuring signal over the sampling time interval $\Delta t=t_{k+1}-t_{k}$, with $n=\frac{t_{n}-t_{1}}{\Delta t}$ being the number of samples taken over the ($t_{1}$,$t_{n}$) interval. Note that the change in $P_{G}$ over the given interval of time is essentially obtained by summing the successive changes in pressure measured using the GasFET over the $t_{k}\to t_{k+1}$ intervals. Furthermore, since the sensitivity of the GasFET depends on the operating point, evaluation of the change in the partial pressure $P_{G}\left(t_{k+1}\right)-P_{G}\left(t_{k}\right)$ over the (k+1)th interval requires that $P_{G}\left(t_{k}\right)$ be known. This implies that the initial pressure $P_{G}\left(t_{0}\right)$ must be determined based on absolute measurements during calibration in order to proceed with the monitoring process.

In a pH-sensitive ISFET, the drain current varies linearly with pH in a Nernstian fashion. That is, for the H^+^−sensitive FET the amperometric sensitivity in the triode region, $S_{A_{lin}}=\left(-2.3)(kT/q\right)g_{m_{lin}}$ is to a first order of approximation independent of pH. Similarly, over small sampling intervals, drain current variations will approximately correspond to changes in the pH, if the product $S_{A_{lin}}\left(\frac{d_{\mathrm{pH}}}{dt}\right)$ is high. Consequently, the drift-free pH response of the ISFET over a given interval of time can be extracted by summing the values of $\Delta I_{D_{lin}}$. Following an equivalent argument as that presented for the GasFET we can assert that $\Delta I_{D_{lin}}≅\Delta I_{D_{\mathrm{pH}}}=S_{A_{lin}}\cdot \frac{d\mathrm{pH}}{dt}\cdot \Delta t$, based on which the change in pH over a given time interval can be expressed as

(24) $$\mathrm{pH}\left(t_{n}\right)-\mathrm{pH}\left(t_{1}\right)=\left(\frac{1}{S_{A_{lin}}}\right)\overset{k=n}{\underset{k=1}{\sum }}\Delta I_{D_{lin}{}_{\left(t_{k}\to t_{k+1}\right)}}$$

where $\Delta I_{D_{lin}{}_{\left(t_{k}\to t_{k+1}\right)}}$, Δt and n have the same definitions as those given in the case of the GasFET. It should be noted that since the sensitivity of the device threshold voltage to pH in an H^+^−sensitive FET is independent of the operating point, changes in pH can be monitored without knowledge of the initial pH of the electrolyte.

### 2.2. ISFET Fabrication

The *n*–channel Si_3_N_4_-gate pH-sensitive ISFET used in the pH monitoring experiment was fabricated based on a metal-gate, *p*-well CMOS process, by removing the metal forming the gate. The substrate material consisted of an *n*-type, (100) silicon wafer whose resistivity lay in the 4–6 Ωcm range. The *p*-well was defined by diffusion using boron as the dopant with a uniform concentration of 10^16^ cm^−3^. Threshold voltage adjustment by ion implantation was not performed. The gate insulator was formed by a 110-nm layer of silicon nitride serving as the pH-sensitive insulator deposited by the LPCVD method over a 50-nm layer of thermally grown silicon dioxide. The source and drain regions were defined by diffusion of phosphorous with a typical doping concentration of 10^19^ cm^−3^. The drawn length and width of the gate were 15 µm and 450 µm, respectively.

### 2.3. Drift Characterization and Measurement of Sensitivity

**Figure 1:**
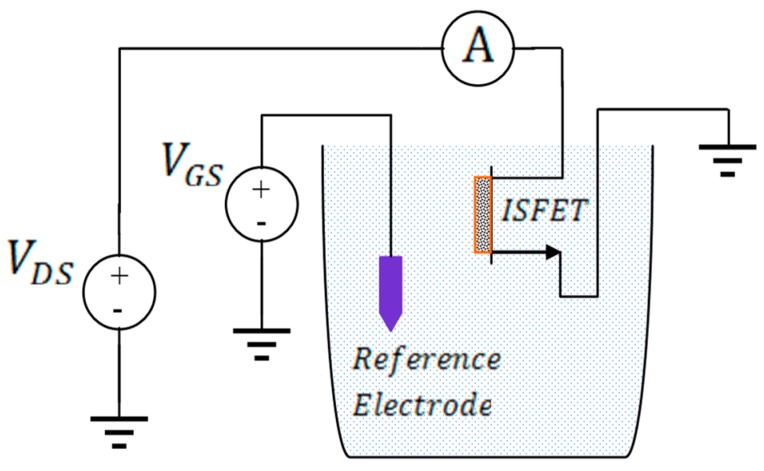
Measurement set-up for characterization of drift in the amperometric mode.

**Figure 2:**
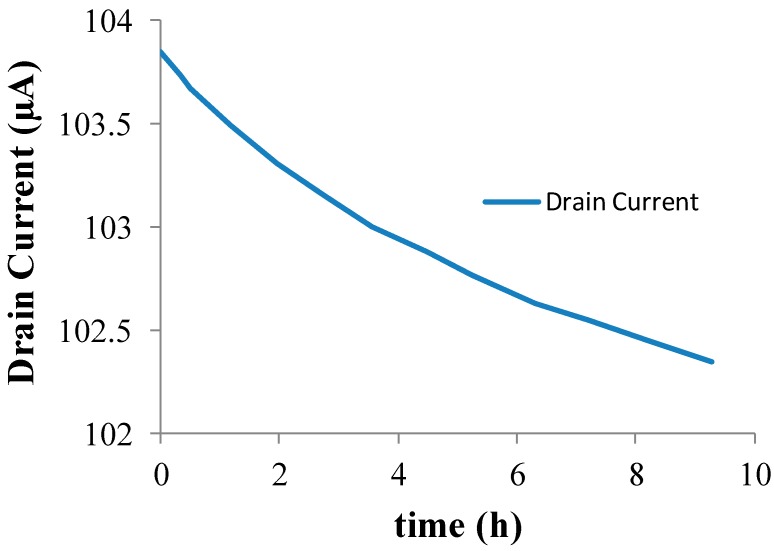
Si_3_N_4_-gate pH-sensitive ion-selective field effect transistor (ISFET) Drift Characteristics at pH = 7.

The drift behavior of the Si_3_N_4_-gate pH-sensitive ISFET used in the pH monitoring experiment was characterized in the amperometric mode at room temperature using the set-up shown in Figure 1. The measured drift characteristics of the Si_3_N_4_-gate pH-sensitive ISFET used for experimental validation of the proposed method is provided in Figure 2. The long-term drift rate for this device after exposure to a neutral buffer solution for 6 h was measured to be −0.0975 μA/h. The ISFET had been placed in the buffer solution for 12 h before conducting the experiment. The threshold voltage of the ISFET was measured to be roughly 1.1 V with the substrate grounded i.e., with no body bias. A gate voltage of $V_{GS}$ = 2.1 V was applied to the solution using a saturated calomel reference electrode to maintain the device in the triode region with the drain voltage set to $V_{DS}$ = 0.2 V.

**Figure 3:**
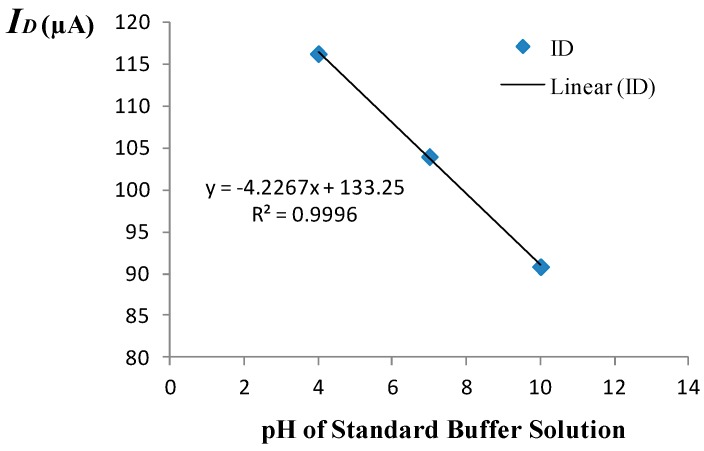
Amperometric calibration curve.

**Figure 4:**
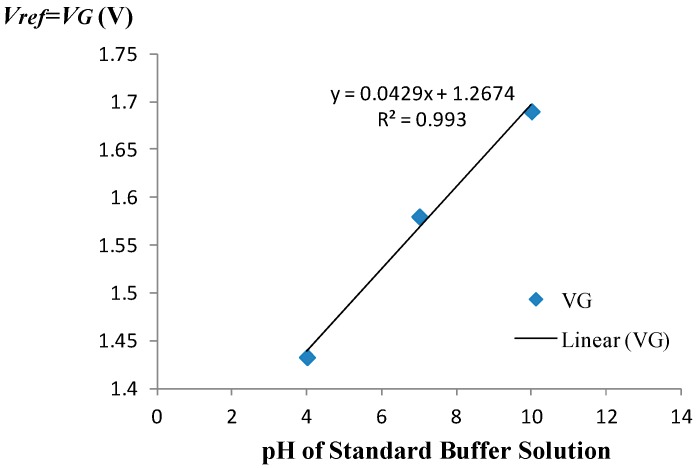
Feedback-mode calibration curve.

The amperometric calibration curve obtained using the measurement set-up of Figure 1 is shown in Figure 3. A 3-point calibration was performed using commercially available standard buffer solutions of pH = 4, pH = 7, and pH = 10. The amperometric sensitivity of the ISFET was determined as the slope of the calibration curve generated by linear regression using standard buffer solutions of known pH based on the mean value of five measured drain current readings recorded at each given pH. Specifically, the amperometric sensitivity in the triode region at room temperature was determined to be −4.23 μA/pH for the device employed to monitor the pH. Prior to use of the device in the monitoring experiment, the equivalent feedback mode sensitivity of the ISFET was determined to be 42.9 mV/pH at room temperature based on the 3-point calibration curve depicted in Figure 4. The measurement set-up used for operating the ISFET in the feedback mode is given elsewhere [8].

### 2.4. Continuous Monitoring of *pH*

Solutions having pH values of 3.5, 5.4, 7.0, 9.0, and 10.0 were prepared in five beakers by adding 1 M HCl or 1 M KOH to a neutral solution containing 0.142 M and 0.05 M potassium chloride and phosphate monobasic, respectively. A pH probe manufactured by Corning (New York, USA) was employed to measure the final pH of each solution using a 601A pH meter manufactured by Orion Research (Beverly, MA, USA) whose accuracy is reported to be 0.01 pH unit. The plasma concentration of sodium was approximated using a 0.142 M KCl baseline solution. Given the sensitivity of Si_3_N_4_-gate ISFETs to sodium, potassium was used instead of sodium. The addition of potassium hydroxide led to approximately 50 mOsm change in the final osmolarity.

After device calibration, the ISFET was arbitrarily placed in each beaker over various time intervals. The drain current was measured at 5 s sampling time intervals in order to monitor the pH response. The change in the drain current was recorded over consecutive sampling time intervals until a stable final value was obtained for the given solution. To compute the corresponding change in pH value, using Equation (24), the sum of the changes in the drain current over successive sampling times prior to stabilization was divided by the measured amperometric sensitivity. The ISFET and the reference electrode were manually transferred to each new solution within 30 s. Following transfer of the device to a new solution, no stirring was performed.

## 3. Results and Discussion

**Figure 5:**
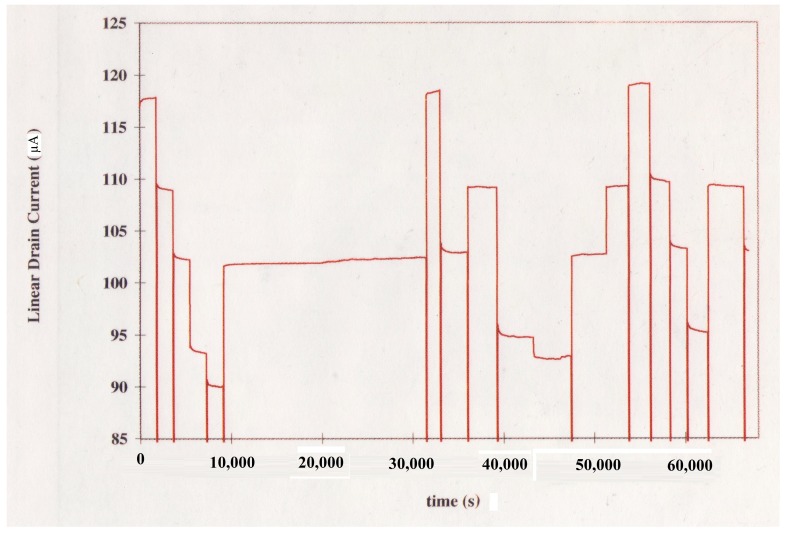
pH Response of the Si_3_N_4_-gate pH-sensitive ISFET.

The pH monitoring experiment performed to validate the method for correction of ISFET drift in the amperometric mode involved application of pH steps in the sequence specified in Table 1. The given sequence of step changes in the pH resulted in the variations of the ISFET drain current shown in Figure 5. The resulting step changes in the drain current following exposure of the ISFET along with the reference electrode to a new solution represent the corresponding experimental pH transitions. Following transfer of the ISFET and the reference electrode to a new beaker, the time required for the pH change to take effect and for the new value to stabilize was generally less than 30 s. The pH step amplitudes measured using the Orion 601A meter were compared, in the order of occurrence of the steps, with the corresponding amplitudes determined with the ISFET based on the proposed corrective scheme using Equation (24). The results of this comparison are provided in Table 1. As indicated, excluding sizeable steps in pH corresponding to changes in pH of 3 units or higher as well as the 9.0→10.0 pH transition, the absolute value of the change in pH as determined based on the proposed method was within 0.2 pH units of that indicated by the pH meter with an average relative error of 5.7%. The relatively larger errors arising in the estimation of large step changes in pH can be ascribed to the hysteresis phenomenon observed as the pH is cycled up and down [14]. Such large variations in pH, nevertheless, are not relevant in the continuous monitoring of physiological pH. The significant inaccuracy in the estimation of the 9.0→10.0 pH transition is due to the considerably larger rate of drift at higher pH values.

As noted in Section 2.1, the accuracy of the proposed method demands a sufficiently high sensitivity. From the measured sensitivity and drift rate for the ISFET used in the monitoring experiment, the condition for the validity of the method expressed by the equivalent of Equation (4) for an ISFET, namely $\frac{\partial I_{D}}{\partial t}\ll \frac{\partial I_{D}}{\partial pH}\cdot \frac{dpH}{dt}$, may be considered to be satisfied if the product $S_{A_{lin}}\frac{dpH}{dt}$ is on average ten times the mean drift rate. Therefore, given the measured drift rate of 0.0975 μA/h at pH = 7 and the sensitivity of −4.23 μA/pH for the ISFET, the minimum acceptable rate of pH change, $\frac{d\mathrm{pH}}{dt}$ guaranteeing the applicability of the method is computed as 10(0.0975)/4.23 = 0.23 pH/h. This indicates that the proposed method is applicable in applications such as cardiac surgery where pH changes at much higher rates, i.e., several tenths of pH unit per minute may be observed. Evidently, the given ISFET does not meet the stability criteria for continuous monitoring during surgery. Nevertheless, introducing a 100-fold increase in the amperometric sensitivity by increasing $g_{m}$ e.g., through upsizing the W/L ratio, an ISFET fabricated in the same technology may be employed to monitor blood pH during surgery, which typically imposes an accuracy requirement of 0.002 pH/h.

The hydrogen-sensitive palladium-gate MOSFETs exhibit a drift behavior similar to that of pH-sensitive ISFETs. Specifically, the drift behavior of the H_2_–sensitive GasFET is characterized by a relatively rapid rise in the threshold voltage after exposure of the sensor surface e.g., Pd to the gaseous medium, followed by a long-term drift at a significantly lower rate [2]. It is interesting to note that the time dependence of drift in palladium-gate MOSFETs [2] can be accurately described by the threshold voltage drift model explaining instability in pH-sensitive ISFETs [3,4]. The cause of drift in Pd-MOS may also be speculated to involve dispersive diffusion in the amorphous gate insulator. Therefore, it is reasonable to assume that the proposed method for correction of drift, which has been experimentally validated in the case of pH-sensitive ISFETs, may also alleviate drift in FET-based gas sensors if the requirement given by Equation (4) is met.

The main advantage gained by operating the ISFET in the feedback mode is that the variations in the potential of the reference electrode may represent the variations in the interfacial potential resulting from pH changes. Ideally, the variations in the reference electrode potential would represent a Nernstian response. This advantage, however, is only realized in the absence of variations stemming from drift, supply voltage, and temperature. In practice, a fixed operating point is maintained through application of feedback regardless of the source of variation. The feedback electronics, on the other hand, not only requires a higher component count, but also leads to significant static power dissipation. A typical ISFET measuring circuit employing feedback requires three operational amplifiers and the associated resistors and capacitors. In applications such as telemetry that require low-power integrated sensor systems, therefore, implementation of the feedback electronics may not justify the considerable additional costs involved. Although the measuring circuit employed in the current mode of ISFET operation is considerably simplified, direct application of the bias voltages to the reference electrode and the drain exposes the ISFET to the same sources of inaccuracies as those encountered in the feedback mode of operation. For example, the current-mode sensitivity of the ISFET varies directly as the gate insulator capacitance, the temporal variation of which contributes to the threshold voltage drift [3,4]. Consequently, supply-independent biasing, temperature compensation and correction, or compensation of drift would still be required in the current mode of operation.

The proposed method has also been successfully applied to correct the threshold voltage drift in a Si_3_N_4_-gate pH-sensitive ISFET operating in the feedback mode [8]. In general, according to Equation (4), the validity of the proposed method requires that the product of device sensitivity and the rate of change in analyte concentration be significantly larger than the drift rate. Therefore, for abrupt changes in pH (i.e., large values of *d*pH*/dt*) the proposed method is effective regardless of the mode of ISFET operation. It is noteworthy, however, that while the ISFET sensitivity in the feedback mode is, to the first order of approximation, independent of bias and device geometry, the device sensitivity can be enhanced in an ISFET operating in the current mode by increasing the device transconductance. In either mode of operation, the accuracy of the method can be improved by employing supply-independent biasing techniques and temperature compensation to render the device sensitivity independent of supply voltage and temperature variations.

Aside from the complicated problem of electrical isolation the instability of the operating point has presented a major impediment to development of inexpensive, disposable FET-based integrated sensor systems for in vivo continuous monitoring of pH and blood gases. If the sensor drift is rendered manageable, given the compatibility of FET-based sensors with the cost-effective CMOS IC technology, a chip integrating ISFETs and GasFETs along with the conditioning circuitry can be readily introduced via a catheter into an artery for monitoring pH and blood gases during surgery. The proposed method for correction of drift involving post-processing of sensor data would require software implementation. However, given the simplicity of the algorithm involved, extraction of the drift-free sensor response is unlikely to lead to unacceptable delays. Nevertheless, ideally the proposed method should be implemented in real-time using CMOS circuit techniques e.g., based on correlated double sampling [15].

## 4. Conclusions

Considering the similarity between the drift behavior of ISFETs and GasFETs, a method for correction of instability in these FET-based sensors was proposed. This method was analytically developed for GasFETs and verified experimentally using a Si_3_N_4_-gate pH-sensitive ISFET operating in the amperometric mode. Irrespective of the mode of ISFET operation, the method was demonstrated to be promising for applications such as continuous monitoring of plasma pH, in which changes in pH are within physiological limits. The proposed method can be generally applicable to correct sensor instability, if a drift signal is superimposed on the measuring signal. Nevertheless, the criterion for the validity of this method requires that the product of sensor sensitivity and the rate of change of analyte concentration be significantly larger than the rate of drift in the measuring signal.

## Figures and Tables

**Table 1:** Comparison of Measured Changes in pH.

Transition Order	Measured Changes in pH		
	pH Meter	ISFET with Correction	Relative Error (%)
1(3.5→5.4)	1.90	1.98	4.2
2(5.4→7.0)	1.42	1.60	12.7
3(7.0→9.0)	2.0	1.99	0.50
4(9.0→10.0)	1.0	0.59	41.0
5(10.0→7.0)	−3.0	−2.71	9.67
6(7.0→3.5)	−3.5	−3.70	5.70
7(3.5→7.0)	3.5	3.48	0.57
8(7.0→5.4)	−1.50	−1.48	1.33
9(5.4→9.0)	3.60	3.12	13.3
10(9.0→10.0)	1.0	0.44	56.0
11(10.0→7.0)	−3.0	−2.27	24.3
12(7.0→5.4)	−1.6	−1.52	5.0
13(5.4→3.5)	−1.9	−2.09	5.3
14(3.5→5.4)	1.90	2.07	8.94
15(5.4→7.0)	1.60	1.43	10.6
16(7.0→9.0)	2.0	1.83	8.50
17(9.0→5.4)	−3.6	−3.3	8.30
